# Sleep quality, arousal and pain thresholds in migraineurs: a blinded controlled polysomnographic study

**DOI:** 10.1186/1129-2377-14-12

**Published:** 2013-02-14

**Authors:** Morten Engstrøm, Knut Hagen, Marte Helene Bjørk, Lars Jacob Stovner, Gøril Bruvik Gravdahl, Marit Stjern, Trond Sand

**Affiliations:** 1 Department of Clinical Neurosciences, Norwegian University of Science and Technology, Trondheim, N-7489, Norway; 2 Department of Neurology and Clinical Neurophysiology, St. Olavs Hospital, Trondheim, N-7006, Norway; 3 Norwegian National Headache Centre, St. Olavs Hospital, Trondheim, N-7006, Norway; 4 Department of Neurology, Haukeland University Hospital, Bergen, N-5021, Norway; 5 Department of Clinical Medicine, University of Bergen, Bergen, N-5021, Norway

**Keywords:** Migraine phase, Sleep, Arousal, Pain thresholds

## Abstract

**Background:**

Our aim was to compare subjective and objective sleep quality and arousal in migraine and to evaluate the relationship between sleep quality and pain thresholds (PT) in controls, interictal, preictal and postictal migraine.

**Methods:**

Polysomnography and PT (to pressure, heat and cold) measurements were done in 34 healthy controls and 50 migraineurs. Subjective sleep quality was assessed by sleep diaries, Epworth sleepiness scale, Karolinska sleep questionnaire and Pittsburgh sleep quality index. Migraineurs who had their sleep registration more than 48 h from an attack were classified as interictal while those who were less than 48 h from an attack were classified as either preictal or postictal.

**Results:**

Migraineurs reported more insomnia and other sleep-related symptoms than controls, but the objective sleep differences were smaller and we found no differences in daytime sleepiness. Interictal migraineurs had more awakenings (p=0.048), a strong tendency for more slow-wave sleep (p=0.050), lower thermal pain thresholds (TPT) (heat pain thresholds p=0.043 and cold pain thresholds p=0.031) than controls. Migraineurs in the preictal phase had shorter latency to sleep onset than controls (p=0.003). Slow-wave sleep correlated negatively with pressure PT and slow bursts correlated negatively with TPT.

**Conclusion:**

Lower PT in interictal migraineurs seems related to increased sleep pressure. We hypothesize that migraineurs on the average suffer from a relative sleep deprivation and need more sleep than healthy controls. Lack of adequate rest might be an attack-precipitating- and hyperalgesia-inducing factor.

## Background

Migraine and sleep are related, though the pathophysiological significance is unclear. Sleep problems are reported by many migraine patients [1]. Sleep related symptoms like tiredness and yawning are particularly frequent in the premonitory phase before an attack [2] while insufficient sleep may trigger a migraine attack [3]. There is also a growing body of neurophysiological evidence suggesting that CNS-excitability changes take place in the preictal state [4,5].

However, there are few polysomnographic (PSG) studies on migraine patients. For patients with sleep-related migraine it has been found that high-frequency arousals are reduced the night before a migraine attack compared to the interictal phase [6]. If disturbed sleep induces, or is related to the triggering of migraine attacks, decreased sleep quality should be consistently found in the preictal phase. If, on the other hand, the headache itself is the main cause for the disturbed sleep in migraine, we would expect decreased sleep quality to be found mainly in the postictal period. It should accordingly be useful so compare objective PSG sleep quality between interictal, preictal and postictal periods in migraine patients.

The relationship between poor sleep quality and migraine pain is still incompletely understood. Reduced pain thresholds (PT) have been found during [7] and before [5] a migraine attack. Since sleep loss and REM sleep loss tend to induce increased pain sensitivity in healthy people [8], it can also be hypothesized that a disturbed sleep structure can cause the observed pre-attack hyperalgesia in migraineurs.

Since the association between subjective and objective sleep quality and the relationship between sleep, migraine cycle and pain physiology is incompletely known in migraine, we intended to study these relationships in a blinded and controlled study design. Our main aim was to compare subjective and objective sleep quality variables and PT in headache free controls and migraine patients in interictal phase. Secondly, we wanted to evaluate PSG sleep quality and PT in interictal, preictal and postictal phases. Thirdly, we intended to perform an explanatory correlation study to enlighten the association between sleep parameters and PT in controls and migraineurs in the three phases.

## Methods

### Participants

One-hundred and twenty six persons, 85 women and 41 men, (age range 18 to 64, mean age 38.9 years), participated in the study. 24 participants with tension type headache will be reported in another paper. They were mainly recruited by advertising in local newspapers for people between 18 and 65 years with- and without headache. Volunteers were examined by a headache specialist who diagnosed patients according to the ICDH-II criteria [9]. Subjects with two to six episodes per month of migraine (M), with- (MA) and without aura (MwoA) were selected. Based on the headache diary (see below), the sleep recordings were divided into interictal, preictal (48 h before the next attack), and postictal (within 48 h after the previous attack). A cutoff of 48 h was chosen to retain power in the interictal vs. control group comparisons, and because the vast majority of reliable premonitory symptoms do not occur until about 24 h before the attack [10]. Three migraine patients with midictal PSG, fulfilling both preictal and postictal criteria, were excluded from this analysis.

Subjects with coexisting frequent migraine and tension-type headache (TTH), other major health problems (sleep disease, hypertension, infection, neoplastic disease, neurological disease, CNS- implants, cardial- or pulmonary disease), chronic or acute pain, regular use of neuroleptic-, antiepileptic- or antidepressant drugs, analgesics, or drugs for migraine prophylaxis the last four weeks), or subjects who were pregnant, were not included. Painkillers or triptans for acute migraine were allowed.

Thirty-four headache-free controls and thirty-three migraine patients in interictal phase, nine in the preictal and eight in the postictal phase were available for analysis in the present study (Table 1). MA and MwoA patients were combined because few preictal and postictal patients were available.

**Table 1:** Background and headache-related data for participants in the present study: Counts or mean (SD)

	**C (n=34)**	**INT (n=33)**	**PRE (n=9)**	**POST (n=8)**
Age (years)	39.6 (13.7)	36.4 (12.9)	41.7 (11.3)	41.1 (11.2)
Sex: F, M	20, 14	25, 8	6, 3	7, 1
BMI (kg/m2)	25.3 (3.4)	23.5 (3.1)	26.2 (4.8)	24.1 (2.8)
Coffeinated beverages (cups per day)	4.0 (3.5)	2.3 (2.3)	4.2 (3.0)	3.8 (2.6)
Alcohol: 0 (never) to 5 (4 or more per week)	**2.9 (1.0)**	2.2 (1.2)**	2.1 (1.2)	**1.8** (1.2)*
Nicotine: yes, no	6, 28	28, 5	6, 3	7, 1
Days since last menstruation	18.9 (8.6)	14.2 (7.6)	15.0 (13.9)	13.8 (10.1)
Married or common-law partner, single	25, 9	19, **14**	9, **0**^#^	7, 1
HAD depression score (0–21)^1^	1.6 (2.1)	2.5 (2.5)	2.1 (2.0)	2.1 (2.0)
HAD anxiety score (0–21)^1^	**2.9 (2.6)**	5.7 (3.0)***	4.7 (2.7)*	6.1 (4.4)
MA, MwoA	na	9, 24	2,7	3, 5
Migraine time in diary (h/day)	na	1.4 (1.9)	1.5 (1.4)	2.1 (1.4)
Headache frequency (1–4)	na	2.2 (0.8)	2.2 (0.4)	2.3 (0.7)
Headache intensity (1–4)	na	2.6 (0.5)	2.2 (0.7)	2.5 (0.5)
Migraine duration (years)	na	20.0 (15.1)	24.2 (10.7)	23.3 (12.4)
Photophobia (0–2)	na	1.1 (0.3)	1.2 (0.7)	1.4 (0.5)
Phonophobia (0–2)	na	1.0 (0.5)	0.9 (0.8)	1.3 (0.7)

*C*: controls. *INT*: Interictal migraine, *PRE*: Preictal migraine. *POST*: Postictal migraine. *C-INT, INT-PRE and INT-POST* differences were all non-significant. *na*: not available. Mann–Whitney *U*-test): *p<0.05, **p<0.005 ***, p<0.001 (in comparison to controls) ^#^ p<0.05 (in comparison to interictal group). ^1^Sum of seven questions about depression and anxiety symptoms respectively during the last week from the Hospital Anxiety and Depression Scale questionnaire.

Significant differences are emphasized with bold types.

The study was approved by the regional ethics committee and participants signed an informed consent before inclusion.

### Questionnaires and diaries

All participants filled in sleep and headache diaries two weeks before and two weeks after the sleep registration. From diaries, the average sleep-time (day and night), awakenings per night, and headache days were calculated. Individual averages were calculated for each subject. For each night sleep latency was (categorized as 0: <15 min, 1: 15–30 min, 2: 31–90 min, 3: > 90 min), long (≥30 min) and short (<30 min) and the average for 14 days was calculated. For sleep latency dichotomous variables (0: < 30 min, 1: ≥30 min) was also analyzed.

Every subject answered several questionnaires including Epworth sleepiness scale (ESS) [11], questions adapted from Karolinska sleep questionnaire (KSQ) [12], Pittsburgh sleep quality index (PSQI) [13] and Hospital anxiety and depression subscales (HADS) [14]. The nine PSQI questions (indicating the frequency of common sleep problems; 0–3) were summed into a combined global score variable (PSQIgs, possible range 0–27) [13]. “Bothersome tiredness” was categorized by as: none, less than 7 days per month, 7–14 days, > 14 days per month, daily (0–4). HADS depression and anxiety subscores, each based on seven of the 14 questions, were calculated. Every subject also quantified their usual pain intensity, the length of their usual headache attack, and scores for photophobia and phonophobia in addition to migraine history duration (Table 1).

### PSG

Patients and controls underwent a full night ambulatory sleep study unattended in our patient-hotel. The hotel is very close and connected by an indoor walking bridge to our department. PSG was recorded by a Notta recorder (EEG Technology Int.bv, 6092 NM Leveroy, The Netherlands) and analyzed with Stellate Harmonie software (Stellate, Montreal, Quebec, Canada). Eight EEG electrodes were placed according to the International (10–20) system [15] (F3, F4, C3, C4, P3, P4, O1, O2 plus Pz reference and Cz ground); two electrooculografic electrodes (EOG) applied two cm laterally and, respectively, two cm above and below the right and left lateral eye cantus. EOG-reference electrodes were applied to the left (A1) and the right (A2) mastoids. Surface electromyography (EMG) was registered from the submental and left anterior tibial muscle.

The following sensors were also applied for respiration and circulation measurements: a three-point oronasal airflow thermistor, a snore microphone, bands around thorax and abdomen to measure respiratory movements (Ultima Respiratory Effort Sensor™, piezo-electric crystals, Breabon Medical Corporation, Carp, Ontario, Canada), a body position sensor, (Ultima Body Position Sensor™, Breabon Medical Corporation, Carp, Ontario, Canada), an infrared index finger oximeter, and two ECG electrodes. The participants were instructed to go to bed, sleep as normal and write down lights-off and lights-on time from a synchronized watch.

Fifteen sleep recordings (seven controls, eight migraine patients) were excluded for technical reasons such as battery error or lost electrodes.

### PSG data analysis

Analyses were done from noted time for “lights off” in the evening to “lights on” in the morning. Sleep stage percentages N1, N2, N3 (SWS=“slow-wave sleep”), REM, arousals and respiratory events were visually scored. Automatic analysis was applied for leg movements. Sleep staging was performed according to “The AASM Manual for the scoring of sleep and associated events” from 2007 [16] with a few exceptions, as described below.

Fast and slow arousals were scored separately. First fast arousals were defined according to the AASM-manual [16] as an abrupt shift of EEG frequency (alpha, theta and/or faster than 16 Hz activity) lasting 3–30 s, separated with at least 10 s of sleep. Arousals were scored in NREM and in REM sleep if associated with increased EMG for more than one second. Although the upper limit for arousal definition is not defined by AASM, we chose to use 30 s in the present study to avoid ambiguous counts induced by random timing of sleep staging epochs. In this way, an e.g. 25-s EEG-frequency increase will always be counted as one arousal event regardless of its relationship to the epoch boundaries. Therefore, only change in EEG-activity containing dominating frequencies of 8 Hz or more lasting more than 30 s was classified as an awakening. If a sleep stage N2 K-complex was followed by a high frequency arousal, we scored the arousal without changing the sleep stage to N1. We also recorded two additional PSG measures of slow-wave arousal in NREM-sleep: Delta-bursts (D-bursts), defined as a sequence of delta waves lasting 2 s or more and exceeding the background amplitude with at least one third [17], and K-bursts, defined as at least two consecutive K-complexes [17]. A K-complex is a negative deflection followed by a positive component with a minimum duration of 0.5 s and minimum peak to peak amplitude of 75 μV observable in at least three EEG channels. Awakening-, arousal- K- and D-burst- indexes were calculated as event number per sleep hour. Since K- and D-bursts probably reflect similar physiological processes [18] they were combined into a KD-index in the present paper. Scoring of respiratory events was first done automatically by the Stellate Harmonie software (Stellate, Montreal, Quebec, Canada). Manual sleep scoring, arousal scoring, and event editing was performed by the first author (specialist in clinical neurophysiology) assisted by a sleep expert (the last author).

### Pain thresholds (PT)

Thermal PT and pressure PT (algometry) were recorded one hour before the participants had their PSG equipment mounted. Heat and cold PT (HPT and CPT) were measured separately in a fixed order on thenar and the medial forehead on both sides with methods of limits (MSA Thermotest, thermode area 25× 50 mm^2^, Somedic Sales AB, Sweden). Temperature was increased by 1°C/s from a 32°C baseline to a 50°C maximum for three HPTs followed by three decreasing temperature stimuli to 5°C minimum for CPT. Pressure PT (PPT) were measured at four sites on both sides in a fixed order: m. temporalis (10 mm lateral to the external angle of the orbit), m. splenius (C2 level just at the edge of the trapezius muscle about 35–40 mm lateral to the midline), m. trapezius (10 mm lateral to the midpoint of a line connecting the acromion and the spinous process of C7) and over distal phalanx middle finger (Algometer type II, probe area 1 cm^2^, Somedic Sales AB, Sweden). Pressure was increased with 30 kPa/s. Thresholds were repeated three times, left before right, and the average was calculated. All thresholds were measured by one out of two technicians. In subjects who did not feel cold pain at 5°C, we used the substitution value 4°C. Thermal PT were expressed as differences from baseline: HPTd (HPT-32) and CPTd (32-CPT) and averaged (right and left sides from all recorded sites) for the present analysis.

### Blinding

Technicians and scorers of PSG and pain thresholds measurements were blinded for diagnoses. Two nurses administered the participant appointments and questionnaires. They also accompanied the participants to the technicians after having instructed the participant not to tell anything that could reveal their headache trait or state.

### Statistics

Statistical analyses were performed with PASW statistics v.18 and SYSTAT version 11. Univariate two-group comparisons were made by nonparametric Mann–Whitney tests. Categorical data were analyzed with Pearson chi-square test or Fisher’s exact test if any cross tab cells had expected count less than five. The primary univariate comparisons were 1) between controls and interictal migraine patients and 2) between interictal and preictal and between interictal and postictal subgroups. After square root transformation, age-adjusted partial correlation coefficients were calculated while four ANOVAS (PSQI, insomnia, tiredness and pain related sleep problems as dependent variables) were performed to check if the group difference between MIG and CO remained when adjusting for relevant confounders (anxiety, depression, PLM, and AHI). Post hoc Spearman’s rho correlations were calculated between awakening index, stage N3 and fast arousals. Two-sided p-values less than 0.05 were regarded as significant.

The power in t-tests to detect large effect sizes equal to 1.0 SD in two-group comparisons between the interictal and preictal/postictal samples were 76% for INT-PRE and 72% for INT-POST. The power to detect medium effect sizes equal to 0.8 SD in the CO – INT two-group comparison was 91%.

## Results

Migraineurs reported a higher HADS-anxiety score and consumed alcohol less frequently than controls. None of the patients in the preictal phase were single (Table 1).

Migraineurs reported significantly more subjective sleep problems. They had more insomnia symptoms, tiredness, global sleep problems (PSQI) and pain-related sleep difficulties compared to controls (Table 2). The difference remained significant in ANOVAs adjusting for relevant confounders (anxiety, depression, PLM, and AHI), for PSQI (p=0.004), insomnia (p=0.002), tiredness (p=0.018) and pain related sleep problems (p=0.02). In the sleep diary they reported significantly more long awakenings than controls while sleep times did not differ. Migraineurs reported longer sleep latency in the dichotomous pathological/non-pathological variable. However, there were no significant differences in sleepiness (daytime hypersomnia) as measured by ESS (Table 2).

**Table 2:** Sleep diary and sleep related symptom mean values (SD) in controls and all migraine patients

	**C (n=34)**	**M (n=53)**
Average diary sleep time (hour)	7.3 (0.8)	7.2 (1.0)
Long awakenings in diary (no)^1^	**0.10 (0.18)**	0.25 (0.36)*
Short awakenings in diary (no)^2^	0.15 (0.24)	0.31 (0.50)
Sleep latency in diary^3,4^	0.40 (0.43)	0.56 (0.61)
Categorized sleep latency in diary (0 ≤ 30 min, 1 > 30 min)^4^	**32, 2**	**37,15#**
Epworth sleepiness scale (0–24)	5.6 (3.1)	6.5 (3.6)
Snoring/apnea KSQ score (0–8)	1.7 (1.6)	1.8 (1.7)
Daytime tiredness frequency (0–4)	**0.68 (0.84)**	1.15 (0.95)**
Insomnia KSQ score (0–16)^5^	**3.4 (2.3)**	6.1 (2.9)***
PSQIgs (0–21)^6^	**3.8 (2.6)**	6.3 (3.2)***
Pain-related sleep trouble (1–4)^7^	**1.3 (0.7)**	1.9 (1.0)**
Restless legs (0–1)^8^	0.1 (0.4)	0.3 (0.5)

^1^Awakenings lasting more than 30 min, ^2^wakenings lasting less than 30 min. ^3^Each day was categorized as 0: <15 min, 1: 15–30 min, 2: 30–90 min, 3: > 90 min and the individual mean was computed .^4^ One sleep latency was not assessable and was excluded. ^5^Sum of four insomnia-questions in KSQ (Karolinska sleep questionnaire), ^6^ Pittsburgh sleep quality index (PSQI) global score, ^7^ Frequency of pain-related sleep problems during the last month from the PSQI questionnaire, ^7^RLS defined as a positive answer to all four obligatory symptom-criteria. # p=0.012, Fisher exact test.: *p<0.05, **p<0.005 ***, p<0.001, Mann–Whitney *U*-test (in comparison to controls).

Significant differences are emphasized with bold types.

A slightly higher awakening index and a strong tendency to more stage N3 sleep and less fast arousals were found in the INT group compared to controls (Table 3). Preictal migraineurs had shorter latency to sleep onset than interictal migraineurs. TPT were lower in interictal migraine compared to controls (Table 3).

**Table 3:** PSG sleep quality and arousal mean values (SD) and pain thresholds for controls and phase-related migraine subgroups

	**C (n=34)**	**INT (n=33)**	**PRE (n=9)**	**POST (n=8)**	**C vs INT differences**	**Phase differences**
Total sleep time (min)	409 (68)	435 (61)	440 (62)	445 (53)		
Sleep efficiency (%)	90 (08)	91 (6)	91 (6)	94 (3)		
Latency to sleep onset (min)	12.8 (14.6)	**10.3 (17.8)**	**2.0 (3.7)**	6.5 (7.9)		INT>PRE**
Awakening index (no/h)	**1.0 (0.6)**	**1.3 (0.7)**	1.2 (0.7)	0.8 (0.6)	INT>C*	
Wake after sleep onset (min)	31 (27)	32 (18)	41 (23)	22 (14)		
Stage 1 (min)	27 (19)	32 (15)	41 (16)	33 (27)		
Stage 2 (min)	197 (47)	201 (44)	198 (46)	195 (77)		
Stage 3 (min)	**86 (31)**	**97 (28)**	99 (35)	108 (41)	INT>C(*)	
REM (min)	99 (26)	106 (35)	102 (36)	108 (28)		
Apnea-hypopnea index (per hour)	2.7 (3.3)	2.4 (3.0)	4.3 (4.9)	0.9 (0.8)		
Fast arousal index (per sleep hour)	**18.3 (5.7)**	**16.3 (9.1)**	13.4 (5.9)	12.3 (5.5)	C>INT(*)	
D-burst index (per sleep hour)	11.8 (8.0)	9.5 (7.5)	10.1 (8.4)	13.9 (9.1)		
K-burst index (per sleep hour)	3.0 (3.8)	3.3 (2.5)	1.9 (1.2)	3.4 (3.2)		
Slow arousal index (per sleep hour) ^3^	14.8 (10.9)	12.9 (9.1)	12.0 (8.6)	17.3 (11.9)		
PPTavg^1^ (kPa)	661 (249)	549 (135)	582 (194)	539 (70)		
HPTavg^2^ (°C)	**13.4 (3.1)**	**11.7 (3.6)**	13.1 (2.3)	13.6 (2.6)	INT<C*	
CPTavg^2^ (°C)	**20.8 (6.3)**	**17.2 (6.9)**	19.5 (5.8)	18.4 (6.3)	INT<C*	

*C*: controls. *INT*: Interictal migraine, *PRE*: Preictal migraine. *POST*: Postictal migraine. Significant differences (*) p=0.05, *p<0.05, **p<0.005 (Mann–Whitney *U*-test). avg: Regional averages from either ^1^splenius, trapezius, temporalis, and index finger (pressure pain thresholds, PPT) or ^2^forehead and palm (heat and cold pain thresholds, HPT and CPT, expressed as differences from the 32°C baseline) registered after noon before sleep. ^3^ The sum of K- and D-burts.

Significant differences are emphasized with bold types.

**Figure 1:**
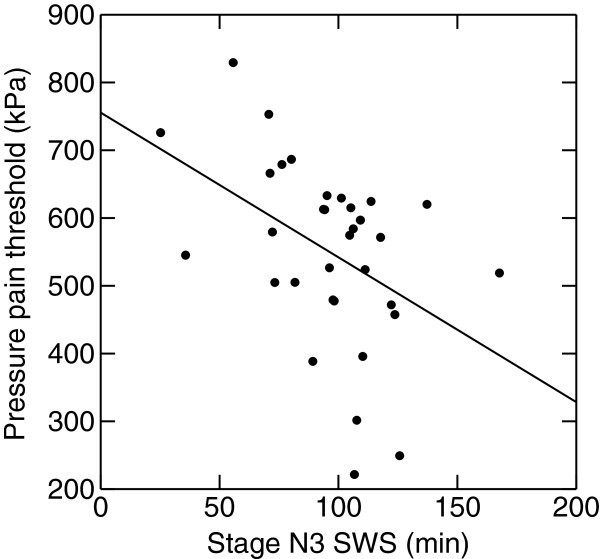
**Pressure pain thresholds and amount of slow wave sleep (SWS) in interictal migraine.** Less N3 sleep is associated with high pressure pain thresholds (age-adjusted r= −0.38, p<0.05).

**Figure 2:**
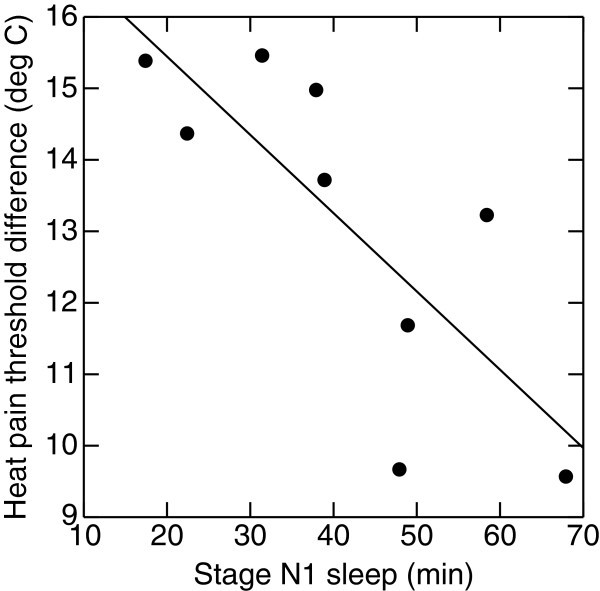
**Preictal migraine subgroup.** High levels of superficial stage N1 sleep is associated with relative heat-pain hyperalgesia (age-adjusted r=−0.80, p=0.02).

Among controls and migraineurs there was a positive partial age-adjusted correlation between anxiety and superficial sleep (Table 4). In interictal migraineurs there was also a negative correlation between SWS and anxiety. Fast arousals correlated positively with insomnia (Table 4). The amount of SWS correlated negatively with PPT (Figure 1) and slow bursts correlated negatively with TPT. Among preictal migraineurs there was a negative correlation between HPT and sleep stage N1 (Figure 2) (p=0.025). In the postictal subgroup REM correlated negatively with CPT (r=−0.79, p=0.03).

**Table 4:** Age-adjusted partial correlations between objective sleep and sleep quality, anxiety and pain thresholds in healthy controls and interictal migraineurs

**Objective sleep**	**Subjective symptoms**			**Pain thresholds**		
**Controls n=34**	**Insomnia**	**PSQIgs**	**Anxiety**	**PPT**	**HPT**	**CPT**
N1 min	0.28	0.15	0.43*	−0.33	−0.12	−0.04
N2 min	−0.12	−0.17	−0.09	−0.20	0.21	0.13
N3 min	−0.27	−0.28	−0.11	0.06	−0.18	−0.13
REM minutes	−0.01	−0.09	0.20	−0.02	−0.14	−0.11
Fast arousals per sleep hour	−0.05	0.12	−0.09	−0.06	0.32	0.31
Slow arousals per sleep hour	−0.06	0.15	−0.31	−0.22	−0.05	−0.08
Interictal						
migraineurs, n=33						
N1 min	0.25	0.35	0.45*	0.15	−0.01	0.14
N2 min	−0.04	0.16	0.43*	0.27	0.05	0.08
N3 min	−0.01	−0.20	−0.37*	−0.37*	−0.23	−0.34
REM minutes	0.12	−0.08	−0.17	0.01	0.21	0.07
Fast arousals per sleep hour	0.43*	0.32	0.16	0.05	−0.18	−0.16
Slow arousals per sleep hour	0.01	−0.15	−0.05	0.06	−0.39*	−0.41*
P*<0.05, P**<0.01.						

Significant differences are emphasized with bold types. *p<0.05.

Migraine history duration correlated (age adjusted) negatively with sleep stage N3 (r=−0.41, p<0.05), while a tendency also was observed for KD-bursts (r=−0.33, p=0.067). We found no significant correlations between our sleep variables and headache frequency or headache intensity.

Post hoc we found a negative correlation in interictal migraineurs between N3 sleep and awakening index (rho=−0.45, p=0.009) and fast arousals (rho=−0.56, p=0.01) (not tabulated).

## Discussion

The main findings in this blinded controlled study were that migraineurs reported more sleep related symptoms than controls while the corresponding differences in sleep diaries and PSG sleep quality variables were much smaller. Interestingly, migraineurs had lower PT and a strong tendency to more N3 sleep. We hypothesize that the latter findings are explained by an increased sleep pressure in the interictal phase.

### Subjective and objective sleep quality in controls and interictal migraineurs

In accordance with other studies migraineurs presently report more subjective symptoms of anxiety [19], insomnia [20] and subjective tiredness [21], than healthy subjects. However, in contrast to these clear differences in subjective symptoms the differences in objective sleep diary variables, daytime hypersomnia scales, and PSG sleep parameters between migraineurs and controls were smaller. A similar discrepancy has previously been found among chronic insomniacs [22]. Insomnia is also a risk factor for migraine [23]. Although patients with known and previously diagnosed sleep disorders were excluded from the present study we can hardly rule out that some migraineurs had an undetected insomnia. Indeed, the high PSQI suggest that some migraine patients may suffer from a sleep disorder. However, PSQI is a summary measure that incorporates insomnia, hypersomnia, snoring and other symptoms and can not be interpreted as a specific sign of insomnia alone. In this study we have focused on sleep differences between our groups, not individualized sleep diagnoses. Furthermore, we excluded those with a clear history of comorbid sleep disorders to characterize the migraine-related sleep dysfunction (including insomnia).

The more objective findings were somewhat inconclusive for insomnia as more migraineurs reported long sleep latencies and long awakenings while PSG sleep latency and total sleep times in both PSG and sleep diaries did not differ from controls.

Despite excessive daytime tiredness among migraineurs we found sleepiness (ESS) to be normal, in agreement with Seidel et al. [24]. However, slightly elevated ESS abnormality rates (ESS cutoff ≥10) in migraine have been observed in a larger population study [1], suggesting that migraine patients on the average achieve somewhat less sleep during nighttime than they actually need.

In the present study both controls and patients with interictal migraineurs had increasing amount of N1 sleep with increasing symptoms of anxiety. However, in the migraine group we also found increasing symptoms of insomnia and decreasing subjective sleep quality with increasing amount of fast arousals. We also found decreasing amount of SWS with increasing amount of anxiety in the interictal migraine group. Hence sleep quality seems to be more vulnerable (i.e. disturbed by anxiety or subjective insomnia) among migraineurs than healthy controls.

In PSG recordings we found almost significant (p=0.05), tendencies towards less fast arousals and more N3 sleep among migraineurs in the interictal phase than among controls. Because fast arousals occur more frequently in superficial than deep NREM sleep [25] it is possible that less fast arousals partly is a passive reflection of more N3 sleep. More deep sleep is one factor that usually indicates better sleep quality [26], but in migraineurs it was paradoxically accompanied by more awakenings. It is possible that awakenings increase sleep pressure and induce compensatory more deep sleep between awakenings. On the other hand, it seems illogic that nights with more awakenings is the explanation for more deep sleep because negative correlations were found between N3 sleep and both awakening index and fast arousals. Another explanation for the paradoxical findings could be subgroup differences. Our findings are only partly in line with findings of Karthik et al. [27]. In that study thirty migraineurs without aura, recruited from a tertiary university hospital, were found to have longer latency to sleep onset, less NREM sleep, lower arousal-index, more awakenings, longer time in bed and more awake time compared to 32 controls. In PSG, we did not find longer latency to sleep onset or less NREM sleep among migraineurs, but we have included migraineurs from advertisement and not hospital patients. Furthermore, Karthik et al. [27] did not say anything about blinding or the temporal relation to the attack.

De Gennaro et al. [28] found reduced amount of fast arousals and increased SWS the night after sleep deprivation. Hence, our PSG findings could indicate sleep deprivation among the interictal migraineurs. However, according to the sleep diaries, sleep deprivation was probably not present in this study, unless one postulates too low power to detect small differences for sleep time in diaries or that migraineurs need more sleep than controls. Even though more SWS is found after sleep deprivation, Sforza et al. [29] did not find more slow bursts while Nicholas et al. [30] found increased numbers of K-complexes per minute in sleep stage 2 after sleep deprivation. A lower number of low frequency, high amplitude EEG bursts in NREM and a lower index of high frequency EEG arousals during REM sleep have previously been found among interictal migraineurs with attacks related to sleep compared to controls [31]. These findings have been interpreted as hypofunction of the arousal system [31], but we suggest that this may be related to a relative sleep deprivation and that migraineurs are relatively sleep deprived and need more sleep than healthy controls.

### Differences in objective sleep among migraineurs in inter, pre and post ictal phases

A shorter latency to sleep onset in the preictal phase compared to interictal phase was the only significant phase difference in PSG variables we found among migraineurs. As far as we know, reduced sleep latency in the preictal phase has not been described before. Hence, preictal migraineurs seem to have greater sleep pressure that fits with premonitory symptoms reported in the preictal phase [10] and daytime EEG changes in the preictal phase [4]. We found no other significant differences in our PSG measures between migraineurs in different phases, probably due to too low power, because we captured only 9 patients in the preictal and 8 in the postictal phase.

Less arousals [6] and more SWS (and also REM sleep) [32] have been found during the night before an attack. We could not confirm these findings in our study. Göder et al. [6] and Dexter [32] included patients with morning- or sleep- related migraine attacks and compared intra individually nights before an attack with nights without ensuing attacks in order to improve the statistical power of their studies. In contrast we compared subgroups inter individually and we also included those with sleep recordings between 24 and 48 h before attack. Because only one of our nine preictal patients had attacks usually initiated by sleep, the study populations are not quite comparable, possibly explaining the discrepant results. As we did not find changes in sleep quality in the postictal group, our result do not suggest that the polysomnographic changes are caused by the attack or sleep disturbances that may accompany the attack in some patients.

### Pain and sleep

We found decreased PT in the interictal migraine group 48 h from any attacks in accordance with Schwedt et al. [33]. However, the latter studies did not exclude preictal migraine from their “interictal” groups. Previously, we did not find significant thermal PT differences between interictal migraine and controls, neither with 24 nor 72 h cutoffs [5], possibly caused by lack of power. The same study showed decreased pain thresholds 24 h before the attack [5] compared to the interictal period. However, the present study was not designed to re-address the latter question as longitudinal observations were not included. Allodynia during a migraine attack has also been described previously [34].

As slow bursts are frequent before and during slow wave sleep, especially in the first sleep cycles [35], these slow bursts are possibly related to sleep pressure or sleep need. Then, both increased N3 sleep and more slow arousals in a stable interictal phase could be assumed to be measures of increased sleep pressure. N3 sleep correlated negatively with PPT and slow burst correlated negatively with TPT in the present study, hence signs of increased sleep pressure seems to be associated with lower pain thresholds. The relationship between PSG signs of a tired (or “weary”) brain and PT does not seem unreasonable as increased pain sensitivity has been found among healthy volunteers after sleep deprivation [8]. Lovati et al. [36] also found in a questionnaire study that migraineurs with head allodynia during attack reported more symptoms of insomnia.

Apparently it seems inconsistent that HPT in the preictal migraine group was negatively correlated to superficial sleep, that is N1 (Figure 2), and not to N3 as in the interictal phase. The reason is unclear, but is should be noted that, compared to controls, interictal migraineurs both had increased SWS and reduced PT. As explained above, we apprehend increased SWS as a probable compensation for increased sleep pressure. Hence N3 could be a measure of both foregoing cerebral tiredness and the ability to compensate for it. As the needs accumulate, in our study indicated by preictally reduced latency to sleep onset in PSG, lack of compensation might induce, or contribute to a migraine attack. When maximum N3 sleep is achieved, any further need for repose could be reflected by the less restful N1 sleep.

### Migraine and sleep

With respect to a hypothesized relative sleep deprivation it makes sense that migraineurs have increased symptoms of tiredness, have either normal EEGs or subtle findings that may reflect drowsiness [37], and tend to have low PT. Even though small undetected differences can not be ruled out, sleep diaries revealed normal sleep times among migraineurs. But why should migraineurs need more rest than controls and how should lack of sleep initiate a migraine attack? More deep sleep may possibly be induced either by increased awake neuronal activity [38] or an absolute sleep deprivation [28]. Since the latter option apparently is not the case here (although a relative deprivation can be operative), increased neural activity might be the link between mental stress (both as state and trait factors (i.e. anxiety symptoms)) and the onset of a migraine attack [39]. Partial correlation adjusted for age indicates that migraineurs have reduced SWS with increasing headache duration. If amount of SWS goes down to a “normal level” with increasing duration of the migraine, this might be a parallel phenomena to reduced migraine attack prevalence after menopause [40].

### Strengths and limitations

The strengths of this study are the blinded, controlled, and prospective design. The participants were mainly recruited by advertising in local newspapers, in contrast to a hospital-based migraine population which may include more severe and longstanding cases. A study design with repeated PSGs may be more powerful for the detection of phase-related differences. In addition, a so-called first-night effect should be considered [41]. Even though a first-night effect has been shown to occur in inpatient PSGs, there are also findings indicating reverse first night effect for some subjects (i.e., increased sleep quality) [42]. Even with two PSGs it is not possible to eliminate the first-night-like effects, because these might last more for than one night in some subjects [41]. In addition, a single PSG design may also have some advantages because serial PSGs can be affected by individually variable order-effects [43]. A slight underestimation of sleep problems, as compared to the general migraine population, is expected in our study because patients with any known and diagnosed previous sleep disorders were excluded, however, this exclusion is a strength regarding the major aims of the study. This study was exploratory and we did not adjust for multiple comparisons because we did not want to increase type II failures on the cost of reducing type I failures [44,45]. Another limitation is the rather low power for comparing interictal, pre- and postictal subgroups and the possibility of both type I and type II errors is acknowledged. Independent replication of our results is accordingly needed.

## Conclusion

In conclusion, migraineurs reported more sleep related symptoms than controls, but the differences in sleep diaries and PSG between the groups were smaller. Migraineurs in the interictal phase had lower PT and tended to have more SWS than controls. Pain sensitivity seems related to increased sleep pressure among interictal migraineurs. Preictal migraineurs also seem to have an increasing sleep pressure. We hypothesize that migraineurs on the average might suffer from a relative sleep deprivation and need more sleep than healthy controls. Lack of adequate rest might be an attack-precipitating- and hyperalgesia-inducing factor. To our knowledge the relationship between sleep and pain has not been investigated in migraineurs before in a blinded, controlled design. For this reason and due to the small number of subjects in the subgroups, the present results should be considered as preliminary and studied further in a longitudinal design.

## Competing interest

The authors declare that they have no competing interests.

## Authors’ contribution

ME mounted some PSGs and performed some pain threshold measurements, analyzed all PSGs, performed the statistical analysis, prepared the initial draft and was the main author of the present manuscript. KH and LJS included patients in the study. GG was contact person for the participants, handled and typed the questionnaires. MS mounted most of the PSGs and performed most of the pain threshold measurements. TS had the original idea of the study; he has made all the data files for statistics and been the main supervisor in all processes. All authors have contributed to the practical plans for the study, read, revised and approved the final manuscript.

## References

[B1] Odegard SS Engstrom M Sand T Stovner LJ Zwart JA Hagen K Associations between sleep disturbance and primary headaches: the third Nord-Trondelag Health Study J Headache Pain 2010 14 3 197 206 10.1007/s10194-010-0201-8 20224943 PMC3451918

[B2] Blau JN Resolution of migraine attacks: sleep and the recovery phase J Neurol Neurosurg Psychiatry 1982 14 3 223 226 10.1136/jnnp.45.3.223 7086442 PMC491341

[B3] Alstadhaug K Salvesen R Bekkelund S Insomnia and circadian variation of attacks in episodic migraine Headache 2007 14 8 1184 1188 10.1111/j.1526-4610.2007.00858.x 17883523

[B4] Bjork M Stovner LJ Hagen K Sand T What initiates a migraine attack? Conclusions from four longitudinal studies of quantitative EEG and steady-state visual-evoked potentials in migraineurs Acta Neurol Scand Suppl 2011 14 56 63 21711258 10.1111/j.1600-0404.2011.01545.x

[B5] Sand T Zhitniy N Nilsen KB Helde G Hagen K Stovner LJ Thermal pain thresholds are decreased in the migraine preattack phase Eur J Neurol 2008 14 11 1199 1205 10.1111/j.1468-1331.2008.02276.x 18795945

[B6] Goder R Fritzer G Kapsokalyvas A Kropp P Niederberger U Strenge H Gerber WD Aldenhoff JB Polysomnographic findings in nights preceding a migraine attack Cephalalgia 2001 14 1 31 37 10.1046/j.1468-2982.2001.00141.x 11298661

[B7] Burstein R Yarnitsky D Goor-Aryeh I Ransil BJ Bajwa ZH An association between migraine and cutaneous allodynia Ann Neurol 2000 14 5 614 624 10.1002/1531-8249(200005)47:5<614::AID-ANA9>3.0.CO;2-N 10805332

[B8] Roehrs T Hyde M Blaisdell B Greenwald M Roth T Sleep loss and REM sleep loss are hyperalgesic Sleep 2006 14 2 145 151 16494081 10.1093/sleep/29.2.145

[B9] Olesen JE The International Classification of Headache Disorders: 2nd edition Cephalalgia 2004 14 Supp 1 9 160 10.1111/j.1468-2982.2003.00824.x 14979299

[B10] Giffin NJ Ruggiero L Lipton RB Silberstein SD Tvedskov JF Olesen J Altman J Goadsby PJ Macrae A Premonitory symptoms in migraine: an electronic diary study Neurology 2003 14 6 935 940 10.1212/01.WNL.0000052998.58526.A9 12654956

[B11] Johns MW A new method for measuring daytime sleepiness: the Epworth sleepiness scale Sleep 1991 14 6 540 545 1798888 10.1093/sleep/14.6.540

[B12] Engstrøm M Ødegård SS Sand T Stovner LJ Zwart JA Hagen K The reliability of a New sleep screening questionnaire for large population-based studies: the third Nord-Trøndelag health study Open Sleep J 2011 14 14 19 10.2174/1874620901104010014

[B13] Buysse DJ Reynolds CF 3rd Monk TH Berman SR Kupfer DJ The Pittsburgh Sleep Quality Index: a new instrument for psychiatric practice and research Psychiatry Res 1989 14 2 193 213 10.1016/0165-1781(89)90047-4 2748771

[B14] Zigmond AS Snaith RP The hospital anxiety and depression scale Acta Psychiatr Scand 1983 14 6 361 370 10.1111/j.1600-0447.1983.tb09716.x 6880820

[B15] Klem GH Luders HO Jasper HH Elger C The ten-twenty electrode system of the International Federation. The International Federation of Clinical Neurophysiology Electroencephalogr Clin Neurophysiol Suppl 1999 14 3 6 10590970

[B16] American Academy of Sleep Medicine W, IL 60154, U.S.A The AASM Manual for the Scoring of Sleep and Associated Events. Rules, Terminology and Technical Specifications 2007 American Academy of Sleep Medicine, Westchester, IL

[B17] Sforza E Jouny C Ibanez V Cardiac activation during arousal in humans: further evidence for hierarchy in the arousal response Clin Neurophysiol 2000 14 9 1611 1619 10.1016/S1388-2457(00)00363-1 10964073

[B18] Halasz P Terzano M Parrino L Bodizs R The nature of arousal in sleep J Sleep Res 2004 14 1 1 23 10.1111/j.1365-2869.2004.00388.x 14996030

[B19] Lanteri-Minet M Radat F Chautard MH Lucas C Anxiety and depression associated with migraine: influence on migraine subjects' disability and quality of life, and acute migraine management Pain 2005 14 3 319 326 10.1016/j.pain.2005.09.010 16289799

[B20] Kelman L Rains JC Headache and sleep: examination of sleep patterns and complaints in a large clinical sample of migraineurs Headache 2005 14 7 904 910 10.1111/j.1526-4610.2005.05159.x 15985108

[B21] Barbanti P Fabbrini G Aurilia C Vanacore N Cruccu G A case–control study on excessive daytime sleepiness in episodic migraine Cephalalgia 2007 14 10 1115 1119 10.1111/j.1468-2982.2007.01399.x 17725651

[B22] Rosa RR Bonnet MH Reported chronic insomnia is independent of poor sleep as measured by electroencephalography Psychosom Med 2000 14 4 474 482 10949091 10.1097/00006842-200007000-00004

[B23] Odegard SS Sand T Engstrom M Stovner LJ Zwart JA Hagen K The long-term effect of insomnia on primary headaches: a prospective population-based cohort study (HUNT-2 and HUNT-3) Headache 2011 14 4 570 580 10.1111/j.1526-4610.2011.01859.x 21457241

[B24] Seidel S Hartl T Weber M Matterey S Paul A Riederer F Gharabaghi M Wober-Bingol C Wober C Quality of sleep, fatigue and daytime sleepiness in migraine - a controlled study Cephalalgia 2009 14 6 662 669 10.1111/j.1468-2982.2008.01784.x 19210514

[B25] Terzano MG Parrino L Rosa A Palomba V Smerieri A CAP and arousals in the structural development of sleep: an integrative perspective Sleep Med 2002 14 3 221 229 10.1016/S1389-9457(02)00009-6 14592211

[B26] Keklund G Akerstedt T Objective components of individual differences in subjective sleep quality J Sleep Res 1997 14 4 217 220 10.1111/j.1365-2869.1997.00217.x 9493520

[B27] Karthik N Sinha S Taly AB Kulkarni GB Ramachandraiah CT Rao S Alteration in polysomnographic profile in 'migraine without aura' compared to healthy controls Sleep Med 2012 14 2 211 214 23232042 10.1016/j.sleep.2012.10.019

[B28] De Gennaro L Ferrara M Bertini M EEG arousals in normal sleep: variations induced by total and selective slow-wave sleep deprivation Sleep 2001 14 6 673 679 11560180 10.1093/sleep/24.6.673

[B29] Sforza E Chapotot F Pigeau R Paul PN Buguet A Effects of sleep deprivation on spontaneous arousals in humans Sleep 2004 14 6 1068 1075 15532200 10.1093/sleep/27.6.1068

[B30] Nicholas CL Trinder J Colrain IM Increased production of evoked and spontaneous K-complexes following a night of fragmented sleep Sleep 2002 14 8 882 887 12489895

[B31] Della Marca G Vollono C Rubino M Di Trapani G Mariotti P Tonali PA Dysfunction of arousal systems in sleep-related migraine without aura Cephalalgia 2006 14 7 857 864 10.1111/j.1468-2982.2006.01122.x 16776702

[B32] Dexter JD The relationship between stage III + IV + REM sleep and arousals with migraine Headache 1979 14 7 364 369 10.1111/j.1526-4610.1979.hed1907364.x 229086

[B33] Schwedt TJ Krauss MJ Frey K Gereau RW Episodic and chronic migraineurs are hypersensitive to thermal stimuli between migraine attacks Cephalalgia 2011 14 1 6 12 10.1177/0333102410365108 20974609 PMC3541827

[B34] Burstein R Cutrer MF Yarnitsky D The development of cutaneous allodynia during a migraine attack clinical evidence for the sequential recruitment of spinal and supraspinal nociceptive neurons in migraine Brain 2000 14 Pt 8 1703 1709 10908199 10.1093/brain/123.8.1703

[B35] Terzano MG Parrino L Smerieri A Carli F Nobili L Donadio S Ferrillo F CAP and arousals are involved in the homeostatic and ultradian sleep processes J Sleep Res 2005 14 4 359 368 10.1111/j.1365-2869.2005.00479.x 16364136

[B36] Lovati C D'Amico D Bertora P Raimondi E Rosa S Zardoni M Bussone G Mariani C Correlation between presence of allodynia and sleep quality in migraineurs Neurol Sci 2010 14 Suppl 1 S155 158 20464610 10.1007/s10072-010-0317-2

[B37] Sand T EEG in migraine: a review of the literature Funct Neurol 1991 14 1 7 22 2055554

[B38] Porkka-Heiskanen T Kalinchuk AV Adenosine, energy metabolism and sleep homeostasis Sleep Med Rev 2011 14 2 123 135 10.1016/j.smrv.2010.06.005 20970361

[B39] Kelman L The triggers or precipitants of the acute migraine attack Cephalalgia 2007 14 5 394 402 10.1111/j.1468-2982.2007.01303.x 17403039

[B40] Brandes JL The influence of estrogen on migraine: a systematic review JAMA 2006 14 15 1824 1830 10.1001/jama.295.15.1824 16622144

[B41] Le Bon O Staner L Hoffmann G Dramaix M San Sebastion I Murphy JR Kentos M Pelc I Linkowsky P The first night effect might last more than one night J Psychiatr Res 2001 14 3 165 172 10.1016/S0022-3956(01)00019-X 11461712

[B42] McCall C McCall WV Objective vs Subjective Measurements of Sleep in Depressed Insomniacs: First Night Effect or Reverse First Night Effect? J Clin Sleep Med 2012 14 1 59 65 22334811 10.5664/jcsm.1664 PMC3266334

[B43] Sforza E Chapotot F Pigeau R Buguet A Time of night and first night effects on arousal response in healthy adults Clin Neurophysiol 2008 14 7 1590 1599 10.1016/j.clinph.2008.03.010 18468950

[B44] Schulz KF Grimes DA Multiplicity in randomised trials I: endpoints and treatments Lancet 2005 14 9470 1591 1595 10.1016/S0140-6736(05)66461-6 15866314

[B45] Perneger TV What's wrong with Bonferroni adjustments BMJ 1998 14 7139 1236 1238 10.1136/bmj.316.7139.1236 9553006 PMC1112991

